# Transcriptome analysis of watermelon (*Citrullus lanatus*) fruits in response to *Cucumber green mottle mosaic virus* (CGMMV) infection

**DOI:** 10.1038/s41598-017-17140-4

**Published:** 2017-12-01

**Authors:** Xiaodong Li, Mengnan An, Zihao Xia, Xiaojiao Bai, Yuanhua Wu

**Affiliations:** Plant Virus Laboratory of Plant Protection College, Shenyang Agricultural University, Shenyang, 110866 China

## Abstract

*Cucumber green mottle mosaic virus* (CGMMV) belongs to the *Tobamovirus* genus and is a major global plant virus on cucurbit plants. It causes severe disease symptoms on infected watermelon plants (*Citrullus lanatus*), particularly inducing fruit decay. However, little is known about the molecular mechanism of CGMMV-induced watermelon fruit decay. For this study, comparative analysis of transcriptome profiles of CGMMV-inoculated and mock-inoculated watermelon fruits were conducted via RNA-Seq. A total of 1,621 differently expressed genes (DEGs) were identified in CGMMV-inoculated watermelon, among which 1,052 were up-regulated and 569 were down-regulated. Functional annotation analysis showed that several DEGs were involved in carbohydrate metabolism, hormone biosynthesis and signaling transduction, secondary metabolites biosynthesis, and plant-pathogen interactions. We furthermore found that some DEGs were related to cell wall components and photosynthesis, which may directly be involve in the development of the symptoms associated with diseased watermelons. To confirm the RNA-Seq data, 15 DEGs were selected for gene expression analysis by qRT-PCR. The results showed a strong correlation between these two sets of data. Our study identified many candidate genes for further functional studies during CGMMV-watermelon interactions, and will furthermore help to clarify the understanding of pathogenic mechanism underlying CGMMV infection in cucurbit plants.

## Introduction

*Cucumber green mottle mosaic virus* (CGMMV) is a member of the *Tobamovirus* genus in the *Virgaviridae* family and is an important viral pathogen of cucurbit crops worldwide^1–4^. CGMMV usually produces typical mosaic patterning on the leaves of infected plants and causes fruits deformation^5,6^, and ultimately results in yield reduction and considerable economic losses^1,7,8^. CGMMV was first reported from the UK in 1935^9^ and has subsequently rapidly spread to several countries, including Greece^10^, Japan^11^, Korea^12^, Israel^1^, Pakistan^13^, India^14^, America^15^ and Canada^16^. In China, the occurrence of CGMMV in watermelon-planting areas was first reported in 2006^17^ and has now been found in many provinces and regions across China. It usually causes severe disease symptoms on infected watermelon plants, especially inducing fruit decay, which is also called ‘blood flesh’, where the inner pulp transforms to water-soaked dirty red and even flesh acidulated^4,11,18^. China is the largest watermelon producer of the world and occupied almost 80% of the total global watermelon production in 2014 (http://faostat.fao.org/). Considering the potential threat to the production of cucurbit crops, in May 2007 CGMMV has been listed as a quarantine pest by the Chinese government^19^. Many researches studied disease surveys, molecular detection, and sequence analysis of CGMMV on watermelons^11,12,17,20^. However, the molecular mechanism of watermelon fruit decay caused by CGMMV still remains unclear.

High-throughput sequencing techniques, such as RNA-Seq, provide a powerful tool to investigate the global transcriptome changes of plants in response to pathogen infection. Several virus-plant interaction analyses have been performed at the transcriptional level to study both the physiological and metabolic processes variations of infected plants^21–23^. Virus-derived small interfering RNAs (vsiRNAs) profiles of CGMMV-infected cucumber^24^ and different tissues from bottle gourd^25^ have been investigated via high-throughput sequencing, and the results indicated that the characteristics of the vsiRNAs between different host plants or tissues were distinctly different. Eight novel and 23 known miRNAs have been identified that cucumber leaves produced in response to CGMMV infection, which is useful to help elucidate host-pathogen interactions as well as to screen for cucumber resistance genes^6^. Recently, the miRNAs sequencing analysis of watermelon leaves infected by CGMMV revealed that target genes for CGMMV-responsive miRNAs were involved in cell wall modulation, plant hormone signaling, primary and secondary metabolism, and intracellular transport^26^. Furthermore, several transcriptome analyses of watermelon fruits during development and ripening^27,28^ provided meaningful references to further explore the transcriptome profiling of watermelons under biotic stress.

CGMMV is one of the most widely occurring and damaging viruses on watermelon plants other than *Cucumber mosaic virus* (CMV) and *Watermelon mosaic virus* (WMV)^29^; however, fruit decay and acidification symptoms are only observed on CGMMV infected watermelons. The purpose of our study was to investigate transcriptional changes of watermelon fruits after CGMMV infection, and we expected to offer novel insights into the molecular basis of CGMMV on cucurbit plants. In the present study, we have taken advantage of high-throughput RNA-Seq to identify differentially expressed genes (DEGs) in CGMMV-inoculated watermelon fruits. To the best of our knowledge, this is the first transcriptome study to investigate the comparative analysis of transcriptome profiles between CGMMV-infected and healthy watermelon fruits.

## Results

### Symptom development and virus detection

**Figure 1:**
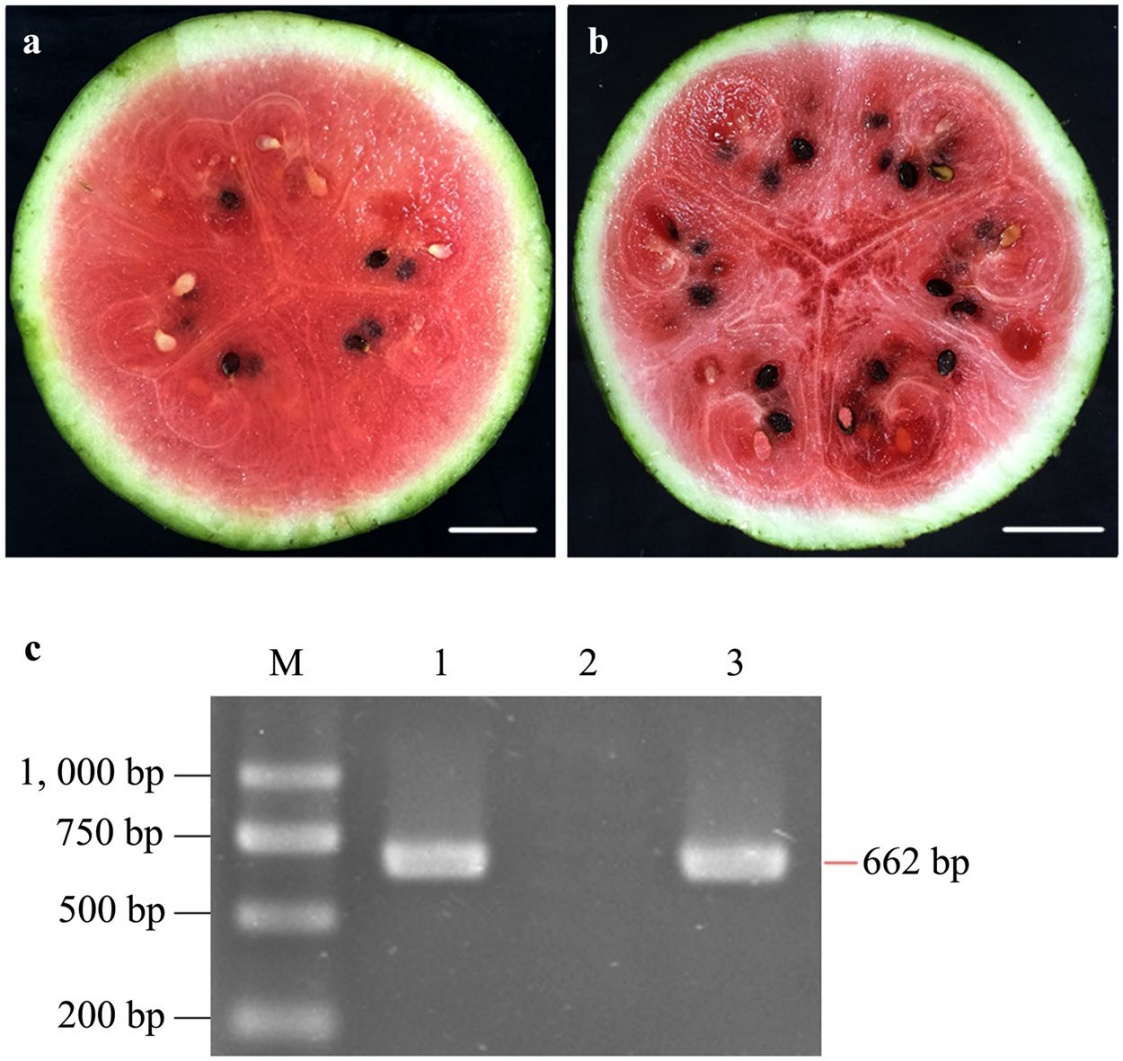
Confirmation of CGMMV infection in watermelon fruit samples (cv. ‘Jingxin No. 3’). (**a**) mock-inoculated flesh; (**b**) CGMMV-inoculated flesh; (**c**) RT-PCR detection results of watermelon fruits with CGMMV specific primers. M: DNA molecular weight marker; 1: CGMMV-inoculated flesh; 2: mock-inoculated flesh; 3: positive control (CGMMV-infected bottle gourd leaves). The full-length gel is presented in Supplementary Figure 1. Scale bar = 5.0 cm.

Watermelon seedlings at the 4-leaf stage were mechanically inoculated with CGMMV, while mock plants were inoculated with virus-free phosphate buffer solution (PBS, pH 7.2) to investigate the changes of host transcriptome. Typical foliar mosaic mottling symptoms on CGMMV-inoculated plants started to appear at 14 days past inoculation (dpi). During the subsequent fruit-maturing period, the inner pulp of the diseased fruits gradually expressed water-soaking deterioration. Mature fruits were harvested and cut into halves. The flesh of CGMMV-inoculated fruits showed severe flesh decay symptoms 32 days after pollination (DAP) (Fig. 1b), while the mock plants showed no symptoms during the entire growth stages (Fig. 1a). The presence of CGMMV in both virus-inoculated and mock-inoculated fruit samples were confirmed by reverse transcriptase-polymerase chain reaction (RT-PCR), in which only the virus-inoculated sample gained PCR products as the positive control (Fig. 1c).

### Global gene expression changes in response to CGMMV infection

Both CGMMV-inoculated and mock-inoculated fruit samples were collected at 32 DAP, and labeled as ‘Treatment’ (CGMMV-inoculated samples) and ‘Control’ (mock-inoculated samples), respectively. Two replicates were conducted in our study and four fruit samples were prepared for RNA extraction followed by Illumina sequencing. Sequencing generated 240,277,630 sequence reads, encompassing about 80.6 Gb of sequence data, which was sufficient for the quantitative analysis of gene expression. After removing low-quality regions, adapters, and possible contamination, the clean reads were obtained with a Q20 percentage over 97.7%, Q30 percentage over 93.9%, and a GC content percentage between 46% and 49%. Then, the clean reads were aligned to the watermelon reference genome database (http://www.icugi.org/cgi-bin/ICuGI/index.cgi)^30^ using TopHat2 software. Of the total reads, 85.35% either matched to a multiple (0.92%) or unique (84.43%) genomic location and the remaining 14.65% remained unmatched (Table 1).

**Table 1:** Illumina sequencing data and the read numbers aligned onto the watermelon reference genome.

Sample name	Treatment 1	Treatment 2	Control 1	Control 2
Raw reads	58,468,222	61,560,586	61,626,230	58,622,592
Clean reads	53,719,928	56,516,432	53,430,956	55,652,612
Total mapped	45,801,815 (85.26%)	48,247,205 (85.37%)	45,817,150 (85.75%)	47,314,915 (85.02%)
Multiple mapped	499,336 (0.93%)	643,348 (1.14%)	598,276 (1.12%)	272,986 (0.49%)
Uniquely mapped	45,302,479 (84.33%)	47,603,857 (84.23%)	45,218,874 (84.63%)	47,041,929 (84.53%)

### Functional annotation and differentially expressed genes (DEGs) classification

**Figure 2:**
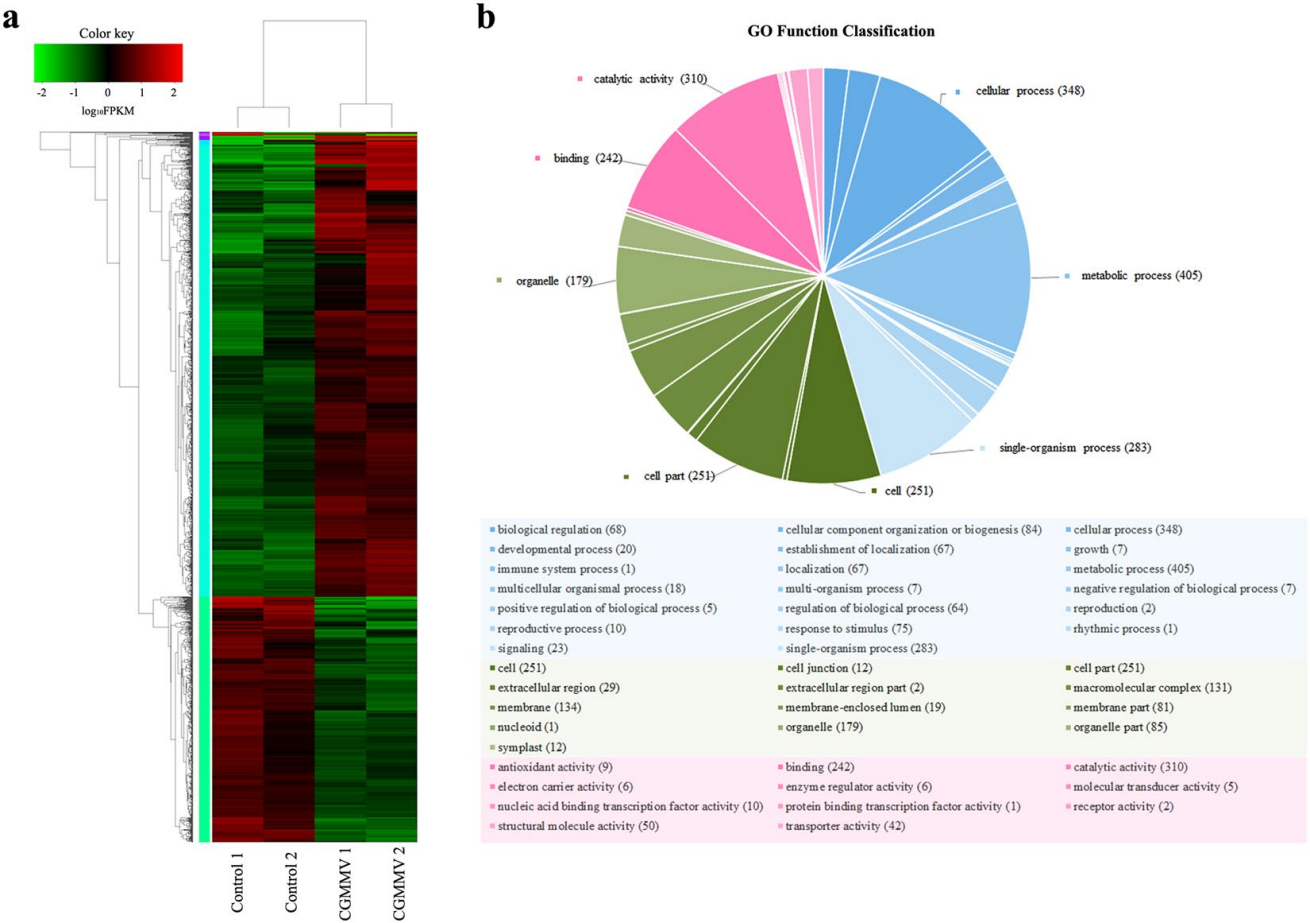
Transcriptome analysis of DEGs in CGMMV-inoculated and mock-inoculated watermelon fruits. (**a**) Hierarchical clustering of DEGs based on log_10_ FPKM values. The color (from green to red) represents gene expression intensity (log_10_ FPKM) from low to high. (**b**) GO function classification of DEGs. The pie chart shows the percentage of DEG numbers of each GO functional group (GO term). Each piece of the pie represents a GO term and all DEGs that belonged to the three main categories (‘biological processes’, ‘cellular components’, and ‘molecular functions’) are filled with blue, green, and pink, respectively. The annotation functions of the top 2/3 numbers of DEGs in each main category are marked in the pie chart.

To identify the watermelon candidate genes which responded to CGMMV infection, four transcriptome profiles were analyzed. Firstly, the expression level of each gene was normalized using the fragments per kilobase of transcript per million mapped reads (FPKM)^31^. Then, the genes with data false discovery rate (FDR) <0.05 and estimated absolute log_2_ fold change (log_2_FC) ≥1 in sequence counts across libraries were considered as significantly DEGs. Finally, a total of 1,621 DEGs were identified between CGMMV-inoculated and mock-inoculated fruit samples. Of these genes, 1,052 genes were up-regulated and 569 genes were down-regulated. To provide an overview of the transcriptome variation, hierarchical clustering was generated and is shown in Fig. 2a.

**Figure 3:**
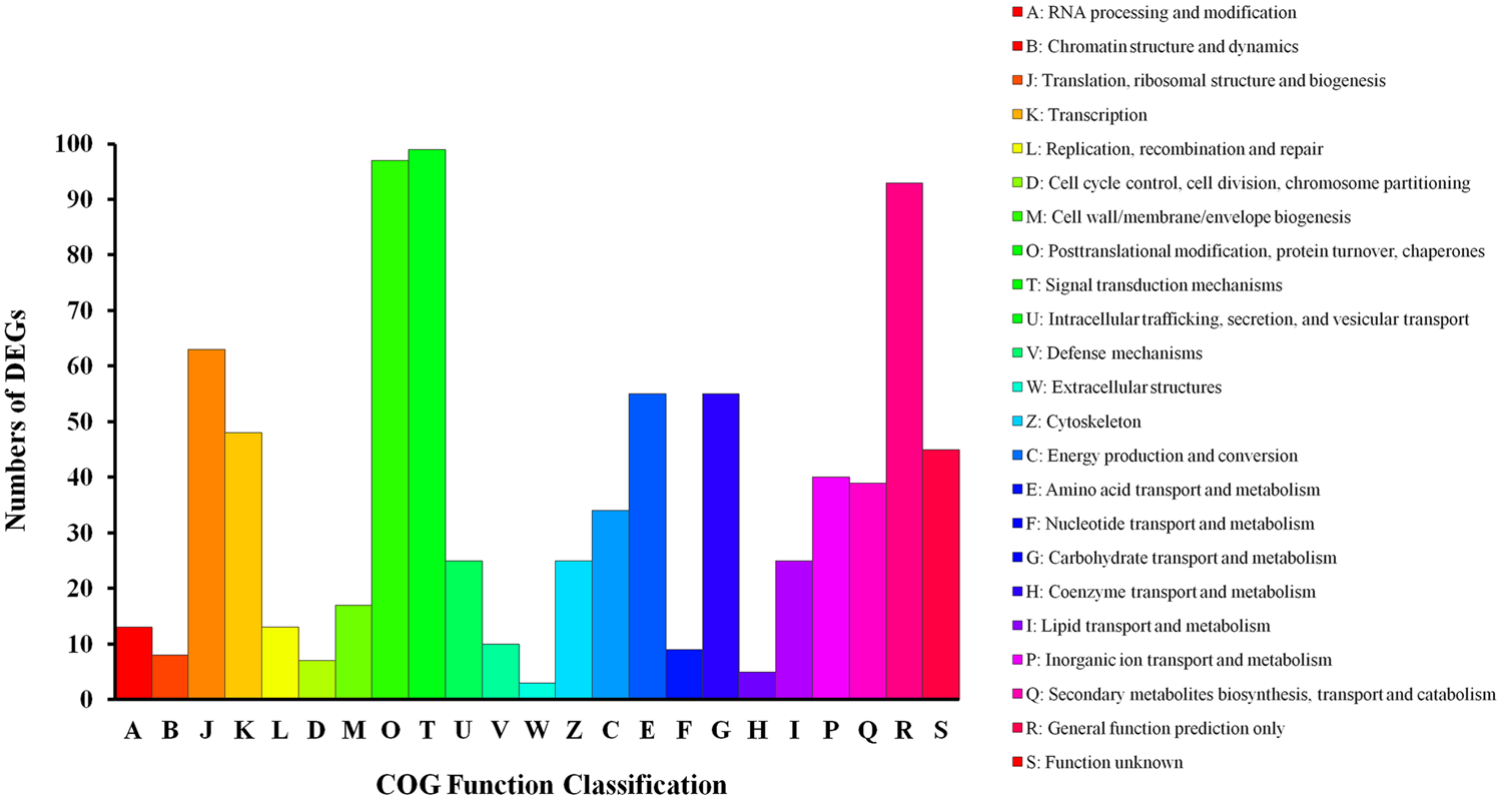
COG function classification of DEGs.

To identify the main functional groups of DEGs, we used a BLASTx search for the NCBI non-redundant protein sequences (Nr), Swiss-Prot, String, Gene Ontology (GO), cluster of orthologous group (COG), and Kyoto Encyclopaedia of Genes and Genome (KEGG) databases. The results indicated that 1,611 DEGs (99.38%) had significant matches within the Nr database, 1,267 (78.16%) within the Swiss-Prot database, 1230 (75.9%) within the String database, 574 (57.82%) within the GO database, 1228 (75.16%) within the COG database, and 709 (43.74%) within the KEGG database. The full list of specific annotation results among the above six databases is shown in Supplementary Tables S1, S2, and S3.The identified functional classes of the DEGs were subjected to a GO enrichment analysis. According to the results of sequence alignments, 574 DEGs could be classified into 43 functional groups (Fig. 2b and Supplementary Table S2) which belonged to three main categories: ‘biological processes’ (1,562), ‘cellular components’ (1,187), and ‘molecular functions’ (683). Most DEGs within the ‘biological processes’ category, were localized to ‘metabolic process’, ‘cellular process’, and ‘single-organism process’. Within the ‘cellular components’ category, ‘cell’ and ‘cell part’ were predominant. The subcategories ‘catalytic activity’ and ‘binding’ were most common in the ‘molecular function’ category. According to the annotation from the COG database, 828 DEGs were grouped into 23 functional categories (Fig. 3). Among these classifications, the clusters of ‘Signal transduction mechanisms’, ‘Posttranslational modification, protein turnover, chaperones’, and ‘General function prediction only’ represented the largest groups, followed by ‘Translation, ribosomal structure, and biogenesis’, ‘Carbohydrate transport and metabolism’, and ‘Amino acid transport and metabolism’. Meanwhile, based on KEGG database, the DEGs were allocated to 296 pathways (Supplementary Table S3). The pathways with the highest number of DEGs were microbial metabolism in diverse environments, starch and sucrose metabolism, and biosynthesis of amino acids. Based on the KEGG pathways enrichment analysis, we found that most DEGs were related with carbohydrate metabolism (ko00500, ko00630, ko00052, and ko00562), amino acid metabolism (ko00500, ko00270, ko00250, ko00480, and ko00260), energy metabolism (ko00190, ko00195, and ko00710), lipid metabolism (ko00564), cellular process (ko04145 and ko04146), and immune system (ko04666) (Table 2). In summary, regardless of the specific databases that were used, most of the DEGs were putatively involved in metabolism processes, signaling transduction, and plant-pathogen interactions. These genes appeared to be relevant for the interactions between watermelon and CGMMV.

**Table 2:** Numbers of DEGs of top 20 metabolism pathways by KEGG enrichment analysis.

Pathway ID	Pathway	Numbers of DEGs (709)	*P*-value
ko01120	Microbial metabolism in diverse environments	41 (5.78%)	0.119022
ko00500	Starch and sucrose metabolism	29 (4.09%)	0.2806
ko01230	Biosynthesis of amino acids	28 (3.95%)	0.163873
ko04141	Protein processing in endoplasmic reticulum	26 (3.67%)	0.098109
ko00230	Purine metabolism	16 (2.26%)	0.388364
ko00190	Oxidative phosphorylation	14 (1.97%)	0.164831
ko00195	Photosynthesis	14 (1.97%)	0.002155
ko00270	Cysteine and methionine metabolism	14 (1.97%)	0.097011
ko00250	Alanine, aspartate, and glutamate metabolism	13 (16.7%)	0.00598
ko00564	Glycerophospholipid metabolism	12 (1.83%)	0.190622
ko00710	Carbon fixation in photosynthetic organisms	12 (1.83%)	0.07612
ko00630	Glyoxylate and dicarboxylate metabolism	11 (14.1%)	0.113511
ko00052	Galactose metabolism	10 (1.69%)	0.088567
ko00480	Glutathione metabolism	10 (1.69%)	0.302041
ko00562	Inositol phosphate metabolism	10 (1.69%)	0.136874
ko00260	Glycine, serine, and threonine metabolism	9 (1.41%)	0.394237
ko00860	Porphyrin and chlorophyll metabolism	9 (1.41%)	0.063637
ko04145	Phagosome	9 (1.41%)	0.298769
ko04146	Peroxisome	9 (1.41%)	0.365976
ko04666	Fc gamma R-mediated phagocytosis	9 (1.41%)	0.31212

### Identification of DEGs involved in carbohydrate metabolism

**Figure 4:**
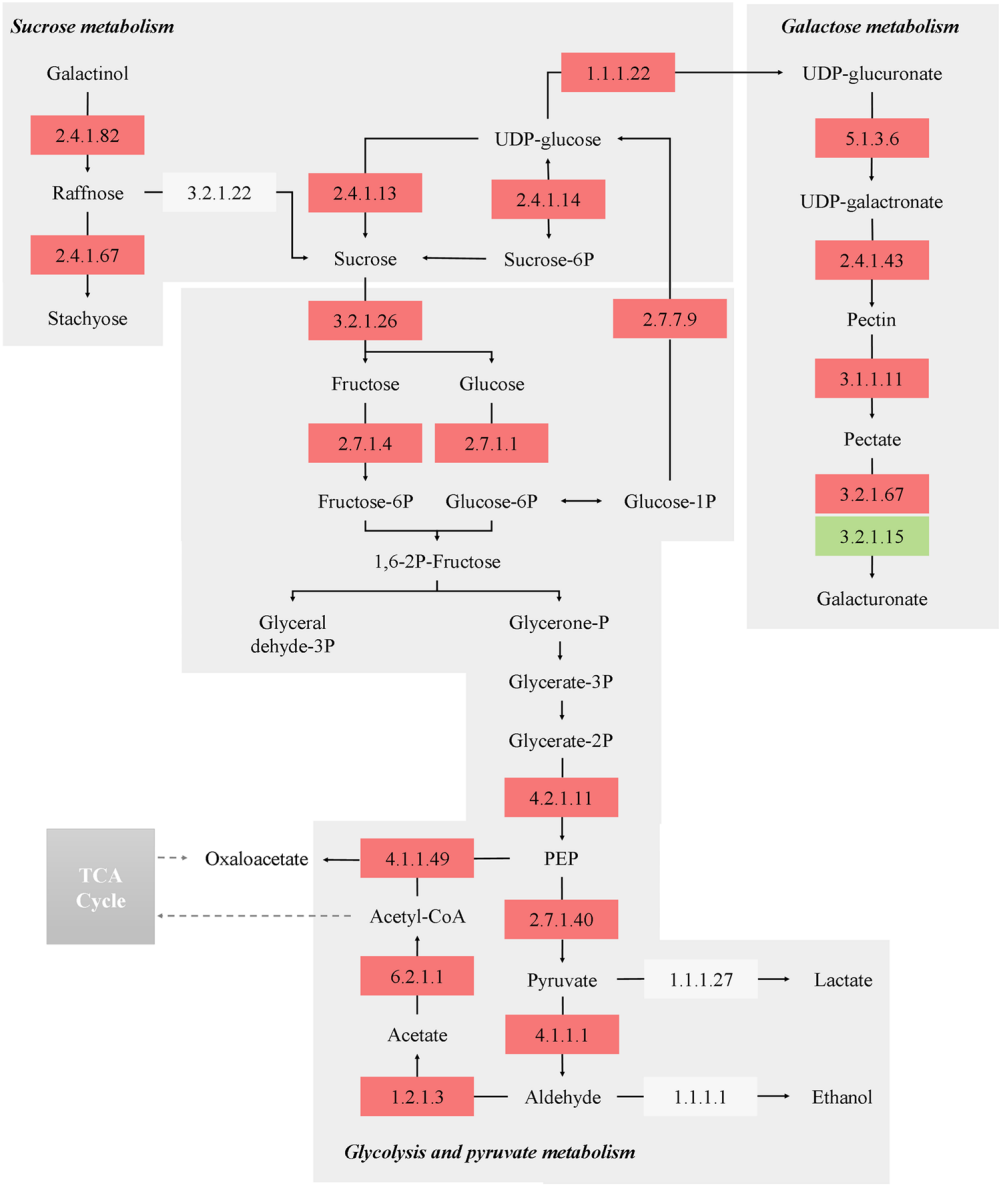
Network diagram of key carbohydrate metabolism-related DEGs of watermelons in response to CGMMV infection. The EC numbers of key enzymes participating in the carbohydrate metabolism are shown in the boxes. The different colors of the boxes indicate that the enzyme encoding genes were up-regulated (red), down-regulated (green), or slightly changed (white), respectively. The list of key enzymes in the diagram was as following: 2.4.1.82, raffinose synthase; 2.4.1.67, stachyose synthase; 3.2.1.22, alpha-galactosidase (GLA); 2.4.1.13, sucrose synthase (SS); 2.4.1.14, sucrose-phosphate synthase (SPS); 1.1.1.22, UDP-glucose 6-dehydrogenase (UGDH); 3.2.1.26, beta-fructofuranosidase (cell wall invertase); 2.7.1.4, fructokinase; 2.7.1.1, hexokinase (HK); 2.7.7.9, UTP-glucose-1-phosphate uridylyltransferase; 4.2.1.11, enolase (ENO); 2.7.1.40, pyruvate kinase (PK); 4.1.1.1, pyruvate decarboxylase (PDC); 1.1.1.27, L-lactate dehydrogenase (LDH); 1.1.1.1.1, alcohol dehydrogenase (ADH); 4.1.1.49, phosphoenolpyruvate carboxykinase (PEPCK); 6.2.1.1, acetate-CoA ligase (ACS); 1.2.1.3, aldehyde dehydrogenase (ALDH); 5.1.3.6, UDP-glucuronate 4-epimerase; 2.4.1.43, alpha-1,4-galacturonosyltransferase (GAUT); 3.1.1.11, pectinesterase; 3.2.1.67, galacturan 1,4-alpha-galacturonidase; 3.2.1.15, polygalacturonase.

Based on COG and KEGG pathways analyses, several DEGs in present study were identified to be implicated in carbohydrate metabolism (Supplementary Table S4). These DEGs mainly participated in starch and sucrose metabolism, glycolysis/gluconeogenesis, galactose metabolism, and pyruvate metabolism. A metabolic network map of these related genes and carbohydrates is shown in Fig. 4. Sucrose synthase, sucrose phosphate synthase, and invertases (also called beta-fructofuranosidase) are the three major enzyme families involved in sucrose metabolism in fruits^32^. The gene expression levels of these three genes (*Cla009124*, *Cla010566*, and *Cla015574*) were significantly up-regulated in CGMMV-inoculated watermelons. We also found three raffinose oligosaccharides (RFO) synthesis enzyme genes (*Cla023372*, *Cla012211*, and *Cla015152*) were highly up-regulated in infected fruits, which may induce an increase of raffinose and stachyose accumulation of fruits. The up-regulated expression of these DEGs may result in the accumulation of sucrose contents in CGMMV-infected flesh and are likely linked to the interaction between CGMMV and the host. The gene expression levels of numerous key enzymes of the glycolysis (such as hexokinase, fructokinase, enolase, and pyruvate kinase) were also distinctly up-regulated in response to the CGMMV infection. In particular, the expression of a HK gene (*Cla002358*) in CGMMV-inoculated fruits was almost three folds higher than that of the control, which may accelerate hexose accumulation in the flesh. In the present transcriptome analysis, we found that the expression of *Cla009675* (a pyruvate decarboxylase gene) was strongly induced in the virus-infected fruits, which may tunnel pyruvate into the ethanol fermentation pathway rather than into the TCA cycle. Meanwhile, some fermentation pathway related enzymes (*Cla009675*, *Cla014544*, and *Cla007147*) exhibited higher expression levels in the CGMMV-inoculated fruits than the mock, which indicated that the anaerobic respiration may also have relations to the watermelon fruits in response to CGMMV infection.

### Identification of DEGs involved in hormone biosynthesis and signaling transduction

Plant hormones play an important role in various defense signaling pathways, and some viruses interact with these signaling pathways to enhance their infection process^33^. Our transcriptome analysis indicated that many DEGs were identified to be related to hormone biosynthesis and signal transduction, including some genes coding for auxin-responsive proteins, auxin-induced protein, gibberellin oxidase, gibberellin-regulated proteins, abscisic acid receptor and ACC oxidases (Supplementary Table S5). Protein kinases have been well-documented to mediate the signaling required for the induction of defense mechanisms, including the activation of transcription factors (TFs) and system responses^34^. Several protein kinases were identified as DEGs in this study, such as leucine-rich repeat receptor-like protein kinases (LRR-RLKs), calcium-dependent protein kinases (CDPKs), mitogen-activated protein kinases (MAPKs), cysteine-rich receptor-like kinases, and serine/threonine-protein kinases (Supplementary Table S5). Transcription factors (TFs) also play crucial roles in the interactions between plants and diverse biotic or abiotic stresses by activating or repressing related downstream genes. The gene expression levels of numerous WRKY, MYBs, basic helix-loop-helix (bHLHs), dehydration responsive element-binding (DREBs), ethylene responsive element binding factor (ERFs) famlies TFs were strongly activated or suppresed by the CGMMV infection.

### Identification of DEGs involved in secondary metabolites biosynthesis

Plant secondary metabolites are well-known to be of importance in plant growth and development, and they also can function as defense molecules, protecting plants in various adverse conditions^35,36^. Results of KEGG pathway analysis showed that dozens of genes, which were significantly activated or suppressed by the CGMMV infection, participated in secondary metabolism pathways. These include phenylpropanoid biosynthesis (ko00940), flavonoid biosynthesis (ko00941), steroid biosynthesis (ko00100), terpenoid backbone biosynthesis (ko00900), anthocyanin biosynthesis (ko00942), and glycerophospholipid metabolism (ko00564) (Supplementary Table S6).

### Identification of DEGs involved in plant-pathogen interactions

In the present study, 13 DEGs have been identified to participate in the plant-pathogen interaction pathway (ko00010), and they mainly encoded respiratory burst oxidase homolog protein, cyclic nucleotide-gated ion channel protein, and calmodulin-like protein. In addition, we also found several DEGs that were related to disease defense and stress resistance functions, including three disease resistance proteins, 21 heat shock proteins (HSPs), three pathogensis-related proteins (PRs), seven glutathione transferases (GSTs), five ankyrin repeat family proteins, one tyrosine aminotransferase, eight protein phosphatases, five peroxidases, one chitinase, and two laccases (Supplementary Table S7). Heat shock proteins (HSPs) included HSP70s, HSP90s, HSP100s, HSP60s, and small heat shock proteins (sHSPs), are stress-responsive proteins that function as molecular chaperones, protecting plants from damage in response to diverse stresses^37^. Notably, the expression levels of 21 HSPs identified in present study were all down-regulated in response to CGMMV infection. PR1s are the most abundantly produced PR proteins in response to pathogen attack. They have been suggested to possess antifungal activity, or to play a role in host salicylic acid (SA)-mediated defense signaling and hypersensitive response (HR)-related cell death^38^. The PR1 gene (*Cla001621*) was strongly induced by CGMMV infection (FPKM >1500) and its expression level in CGMMV-inoculated samples was 3.6 fold that of its counterpart.

### Identification of DEGs involved in symptom development

It was also found that, many DEGs in present study were involved in cell wall formation and photosynthesis (Supplementary Table S8), which could be responsible for the specific symptoms of CGMMV on watermelon fruits. Pectin and cellulose are the major components of plant cell wall, which could be decomposed by cellulose synthase (CES), pectinesterase (PE), and polygalacturonase (PG), respectively. In our data, four PE related genes and six PG related genes were identified as DEGs and most of their expression levels were highly increased in the virus-infected flesh, which might accelerate cell wall disassembly. Otherwise, seven xyloglucan endotransglycosylase (XET) and four xyloglucan synthesis-related genes were identified, and 10 of these positively responded to the CGMMV infection. Eight CES related genes were significantly up-regulated in CGMMV-inoculated flesh, indicating that much higher cellulose contents might accumulate compared to the control. Meanwhile, the KEGG pathway analysis showed that the photosynthesis pathway (ko00195) and porphyrin and chlorophyll metabolism pathway (ko00860) show a certain degree of enrichment, which might be highly associated with the watermelon regulatory network in response to CGMMV infection. In our data, approximately 50 genes which related to photosystem or participated in photosynthesis were identified as DEGs (Supplementary Table S8). The expressions of genes related to functions of chlorophyll, carotenoids, and cytochromes also showed significant alternations in the CGMMV-inoculated plants (Supplementary Table S8).

### Validation of RNA-Seq data by quantitative real-time PCR (qRT-PCR)

**Figure 5:**
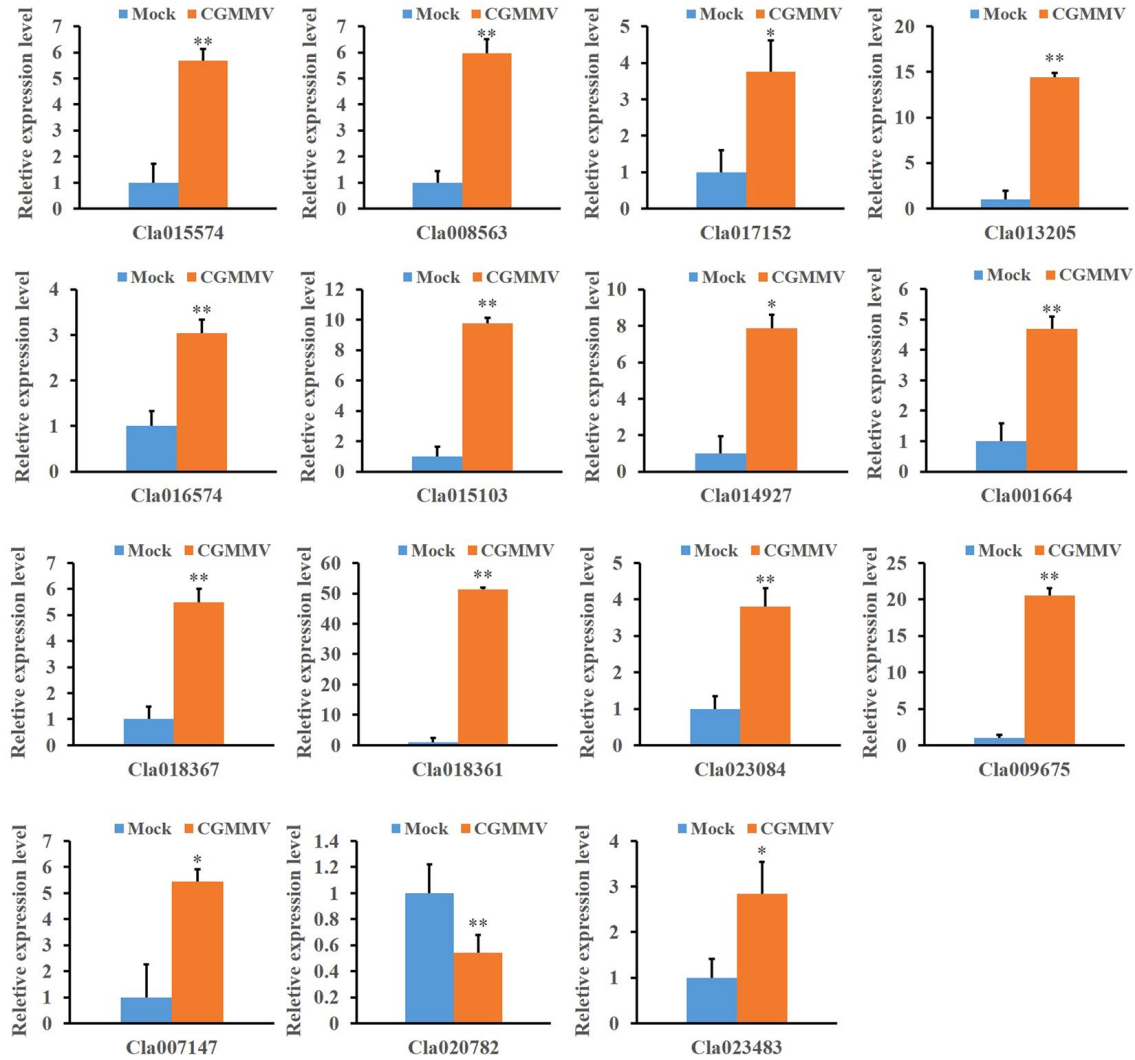
qRT-PCR validation of 15 selected DEGs involved in carbohydrate metabolism in response to CGMMV infection. Informed by the RNA-Seq analysis, 15 DEGs were selected for validation via qRT-PCR analysis. The watermelon 18 S rRNA gene was used as internal control. Error bars represent mean ± standard deviation (SD) and the data are averages of three biological replicates. Asterisks indicate statistically significant differences compared to control (Student’s *t*-test): *P < 0.05, **P < 0.01.

To validate the RNA-Seq data, 15 carbohydrate metabolism-related DEGs were selected for a gene expression analysis via qRT-PCR. The relative expression of these selected genes are shown in Fig. 5. In total, these 15 genes exhibited consistent expression patterns between the RNA-Seq data and qRT-PCR. In general, both data sets strongly correlated in our present study.

## Discussion

Watermelon fruits infected by CGMMV usually showed severe water-soaked decay symptoms, which had significant influences on flesh quality^4^. Sugar content, flesh texture, aroma, and flavor are important factors, which determined the quality of watermelon fruits^39^. The main sugar contents (sucrose, glucose, and fructose) in CGMMV infected watermelons have been determined in our previous study^40^, and the results showed that the virus infection significantly affected these three main sugar contents and the total sugar compositions. Especially, glucose contents of infected flesh accumulated much quicker than in healthy flesh prior to full maturity of the fruit (within 28 DAP), but dramatically reduced when the fruit was harvest (at 35 DAP). Furthermore, the ratios of these three sugars to total sugar were abnormal compared to the mock. In present study, the expression levels of sucrose metabolism-related enzymes (*Cla009124*, *Cla010566*, and *Cla015574*) and three RFOs synthesis enzymes (*Cla023372*, *Cla012211*, and *Cla015152*) were significantly up-regulated which may result in the accumulation of sucrose contents in virus-infected tissues (Supplementary Table S4). In *Cucurbitaceae* family members, RFOs (including raffinose and stachyose), are the predominant photoassimilates translated in the phloem of cucurbit plants^41^. They are also regarded to act as a signaling molecule following pathogen attack and wounding and accumulate in vegetative tissues in response to abiotic stresses^42^. We also found that several key enzymes genes of glycolysis, especially the HK and fructokinase gene, showed the increasing expressions (Supplementary Table S4). A recent review indicated that elevated hexose levels could contribute to the plant defense against viral infection^43^. In addition, the up-regulated expressions of these DEGs were corresponding to the dramatic consumption of glucose in CGMMV-infected flesh as described in our previous study^40^. To investigate the metabolic trends of pyruvate (the final product of the glycolysis) in CGMMV-infected flesh, we previously determined the pyruvate contents and three main pyruvate metabolic contents (lactate, acetaldehyde, and ethanol) of infected samples^44^, in which higher levels of these products were measured compared to the control. As for the transcriptome level, pyruvate metabolism related genes were apparently up-regulated by the CGMMV infection. Generally, pyruvate will generate acetyl-CoA through the pyruvate dehydrogenase complex or produce oxaloacetate (OAA) via pyruvate carboxylase to enter the TCA cycle under aerobic conditions^45^. However, deprivation of O_2_ will trigger the cessation of the TCA cycle and will consequently shift the production of ATP to alcoholic fermentation^46,47^. It has therefore been suggested that plant cells accumulated overly much carbohydrates due to the CGMMV infection, and the temporary deficiency of oxygen derived from the increasing respiration finally resulted in anaerobic metabolism of the infected tissues. The higher expression levels of fermentation pathway related enzymes (*Cla009675*, *Cla014544*, and *Cla007147*) indicated the occurrence of anaerobic respiration in CGMMV-inoculated fruits. The accumulation of ethanol and acetaldehyde in plant tissues is widely known to be harmful for plant development due to cytoplasmic acidosis^48,49^. As a result, the production of these pyruvate metabolites in the infected flesh might severely affect the fruit quality and produce fruits without edible value.

It is known that TFs play a crucial role in the signaling transduction of plants in response to external stimulates. In present study, the gene expression levels of numerous WRKY, MYBs, bHLHs, DREBs and ERFs families TFs were significantly affected by the CGMMV infection (Supplementary Table S5). As one of the largest TF families in plants, WRKYs have been shown to play important roles in plant-pathogen interactions^50^. Several studies revealed that WRKY genes contributed to the regulation of rice response to pathogen infection^51,52^. The AP2/ERF family transcription factors have been reported to be involved in enhancing the tolerances to various abiotic stresses, as well as in the host resistance to viral and bacterial pathogens^53^. Notably, eight ERF genes in our data were strongly affected by viral infection and the resulting expression levels in the CGMMV-inoculated flesh were more than two fold those of the control. Furthermore, four ACC oxidases which related to ethylene biosynthesis showed differential expression levels in response to the CGMMV infection. The *NbACO* gene was also found to be up-regulated in TMV-infected *Nicotiana benthamiana* ^54^. Therefore, it has been postulated that changes of endogenous ethylene contents in CGMMV-infected watermelons could result in the fruits easier decay than the healthy ones. In fact, our previous study showed that the ethylene contents of the infected fruits indeed apparently increased by the CGMMV infection^44^. These results indicated that the ethylene signaling transduction pathway may be related to the host responses to CGMMV infection. Genes coding for auxin-responsive proteins, auxin-induced protein, gibberellin oxidase, gibberellin-regulated proteins, abscisic acid receptor and ACC oxidases were identified as DEGs in the CGMMV-responsive transcriptome profiling of watermelon fruits (Supplementary Table S5). Interestingly, transcripts related to auxin- and gibberellin-regulated, and ethylene biosynthesis functions were also identified from the *Tobacco mosaic virus* (TMV)-infected *N. tabacum* leaves via microarray analysis using tobacco chips^55^. Besides, the expression of *NbARP1* (an auxin-response gene) showed up-regulation in *Hibiscus latent Singapore virus* (HLSV, an attenuated stain of *Tobamovirus*) infected *N. benthamiana* ^54^. Padmanabhan *et al*. also proved that the helicase domain of TMV replicase could interact with a putative regulator of auxin response genes which involved in plant development of *Arabidopsis thaliana* ^55,56^. These findings indicated that the CGMMV infection may influence on the auxin response system and signaling pathway between the CGMMV and watermelon fruits interactions.

In this study, we also found that the expression levels of many host genes which participated in secondary metabolism pathways and plant-pathogen interaction pathway were fluctuated in response to the CGMMV infection (Supplementary Tables S6 and S7). The phenylpropanoid biosynthesis pathway could generate secondary metabolites such as lignin, flavonoids, and anthocyanins, which have been reported to play key roles in the resistance of plants to pathogen infection^57^. Notably, several genes related to the phenylpropanoid biosynthesis pathway were predicted as the targets of CGMMV-responsive miRNAs in watermelon^26^. Flavonoids are a large group of phenolic secondary metabolites that are widely present in plants. Flavonoids have been implicated in a variety of biological functions, such as flower coloring and pollination, defenses against pathogens and insects, and alleviation of oxidative damage^57^. These changes of the secondary metabolism pathways in CGMMV-infected flesh indicated that CGMMV might not only severely affect plant development, but also strongly stimulate the production of plant defensive substances in response to viral infection. HSPs, PRs, GSTs, and several other disease defense related genes were identified as DEGs in present study. Interestingly, in the case of TMV-infected tobacco, several transcripts coding for HSPs, PRs, GSTs, perxiodases, chitinases, and dehydration response factors were similarly induced by the virus infection^55^, which indicated that there may be a commonality between the host transcriptome alterations after different tobamoviruses infection.

Watermelon fruits infected by CGMMV often developed symptoms of malformation, and the inner pulp turned to sponginess, rotting, and dirty red discoloration^4^. Fruit over-softening and water-soaked decay may be due to the damage of the cell wall structure of the flesh. Pectin is the major component of plant cell wall, which could regulate intercellular adhesion; its depolymerization was reported as the main reason for loss of fruit firmness^58^. PE has been demonstrated to play a role in the pectin degradation, as well as in the plant response to pathogen attack. A cell wall-associated PE gene of *N.tabacum* was involved in the host cell receptor recognition of the TMV movement protein and it has been shown that this interaction was required for cell-to-cell translocation of the virus^59^. XET is involved in the synthesis of xyloglucan (the predominant hemicellulose in the cell walls) and it has been reported to play a profound role during fruit ripening^60^. In our comparative transcriptome analysis, several genes coding for PE, PG, and XET were apparently up-regulated by the CGMMV infection (Supplementary Table S8), which suggested that these genes might play central roles in the cell wall solubilization of the diseased watermelon fruits that exhibited over-softening. The up-regulated expressions of eight CES genes could be responsible for the increase of yellow fibers in CGMMV-infected fruits, and further decreasing the taste of the fruits. Watermelons suffering from CGMMV typically exhibit yellow mottling and mosaic on leaves and fruit peels, and sometimes brown necrotic lesions on stems and peduncle^4^. These symptoms could be due to photosystem damages or changes in the chloroplast. We found a lot of DEGs involved in photosynthesis pathway were affected by the CGMMV infection, indicating that these genes were also participated in the interaction networks between CGMMV and watermelon. In addition, differential expressions of photosynthesis-related genes were found in the interactions of host with TMV^55^, *Cucumber mosaic virus* ^61^, *Strawberry vein banding virus* ^22^, and other plant viruses at the transcriptome level.

## Conclusions

In this study, we comparatively analyzed the gene expression profiles of CGMMV-inoculated and mock-inoculated watermelon fruits, using RNA-Seq deep sequencing technology. Our results showed that CGMMV infection affected the gene expression levels of 1,621 DEGs, including 1,052 up-regulated and 569 down-regulated DEGs. Functional annotation of DEGs based on GO, COG, and KEGG pathway analyses showed that these DEGs were mainly involved in metabolic processes, cell components, hormone biosynthesis and signal transduction, plant-pathogen interaction, as well as secondary metabolites biosynthesis. CGMMV infection also noticeably affected the expressions levels of cell wall components and photosystem or photosynthesis related genes, which may be directly involved in the symptom development of the infected watermelons. Our genome-wide transcriptome analysis provides a scientific basis for further investigation of the molecular mechanisms underlying CGMMV infection in watermelons. Further studies should focus on whether and how these pathogenic related genes play essential roles in the interaction between virus and host plants.

## Methods

### Plant propagation and virus inoculation

Watermelon plants (*C. lanatus* cv ‘Jingxin No. 3’) were utilized for this study. The CGMMV virus source (virus isolate: CGMMV-lnxg; GenBank ID: KY040049) was preserved on bottle gourd plants by the Plant Virus Laboratory of the Plant Protection College, at the Shenyang Agricultural University (SYAU), China. Viral sap was prepared from 0.1 g of CGMMV-infected gourd leaves via homogenization with 0.01 mol·L^−1^ PBS for mechanical inoculation of leaves of tested plants at the 4-leaf stage. The mock group plants were inoculated with PBS buffer only. Then, the seedlings were propagated via grafting on virus-free squash rootstocks and transferred to a plastic greenhouse at a melon plantation in Shenyang, Liaoning Province, China (latitude: 41.9511031186; longitude: 122.7212245701). All flowers were hand-pollinated and tagged. Center flesh samples were collected at 8, 16, 24, and 32 DAP for symptoms observation and viral detection of CGMMV in watermelon fruits. To verify successful inoculation, virus-inoculated and mock-inoculated fruits samples were detected via RT-PCR using CGMMV-specific primers (forward primer: 5′-ATGGCTTACAATCCGATCAC-3′; reverse primer: 5′-TGGGCCCCTACCCGGGGA-3′; refer to^17^). Considering that the severe and typical symptoms of CGMMV on watermelon fruits were exhibited at the late maturing period, especially near to the fully maturation time, the fruit samples collected at 32 DAP were prepared for Illumina sequencing. Five individual fruits were pooled as one sample from each group and two replicates were measured in our study. Samples were immediately frozen in liquid nitrogen and stored at −80 °C until further use.

### RNA extraction, cDNA library construction, and Illumina sequencing

Total RNA was extracted from the flesh sample of each group via TRIzol Reagent (Invitrogen, USA) according to the manufacturer’s protocol. The quality and quantity of total RNA was assessed via NanoDrop2000 spectrophotometer (Thermo Fisher Scientific, USA), and RNA integrity was assessed on a 1.5% agarose gel. Purified poly (A) + mRNA was extracted from the total RNA sample using Oligo (dT) magnetic beads. mRNA was fragmented into 200–500 nt pieces by adding a fragmentation buffer. First-strand cDNA was synthesized using SuperScript II reverse transcriptase (Life Technologies, Inc.) and random hexamer primers. After generation of second-strand cDNA, the double-strand cDNA was end-repaired, and a single ‘A’ base and indexed adapters were ligated to the fragments. The cDNA library was constructed via the Illumina Paired End Sample Prep kit (Illumina, USA) and was then sequenced on the Illumina HiSeq. 2000 platform. Sequencing was conducted by the Majorbio Biopharm Technology Co. Ltd. (Shanghai, China). The quality of RNA-Seq was assessed via saturation, duplicate reads, and gene coverage analysis, using RSeQC-2.6.3 (http://rseqc.sourceforge.net/).

### Processing and mapping of Illumina reads

Raw reads generated via Illumina HiSeq. 2000 were initially processed to receive clean reads by removing adapter sequences and low quality reads using SeqPrep (https://github.com/jstjohn/SeqPrep). Then, the remaining high-quality reads were mapped to the watermelon genome using TopHat2 (http://tophat.cbcb.umd.edu/). Mapped reads were used for quantification by RSEM (http://www.biomedsearch.com/nih/RSEM-accurate-transcript-quantificaition-from/21816040.html). To compare gene expression levels between both libraries, the relative gene expression levels were calculated and normalized via FPKM^31^. DEGs were identified via *p*-value < 0.005, FDR < 0.05, and estimated absolute log_2_FC ≥ 1 between both libraries using edgeR software (http://www.bioconductor.org/packages/2.12/bioc/html/edgeR.html). We compared the expression profiles of CGMMV-inoculated treatments to those of the mock-inoculated control to receive expression ratios (fold changes) and respective *p*-value by averaging data of two different biological replicates.

### Functional analysis of DEGs

To determine the functional annotation of DEGs, BLAST alignment was conducted against the following six databases: Nr, Swiss-Prot, String, GO, COG, and KEGG. GO enrichment analysis was implemented in the Goatools program (https://github.com/tanghaibao/GOatools) and KEGG pathway analysis of DEGs was performed with KOBAS (http://kobas.cbi.pku.edu.cn/home.do).

### Validation of RNA-Seq data by qRT-PCR

To verify the accuracy and reproducibility of the Illumina RNA-Seq results, qRT-PCR assays were conducted with gene specific primers. Total RNA was extracted from watermelon fruits using the TRIzol reagent (Invirtrogen, USA) and qRT-PCR was performed using SYBR Premix *Ex Taq* II (TaKaRa, Japan) following the manufacturer’s protocol. qRT-PCR was conducted on an ABI Stepone Real-Time PCR System (Applied Biosystems, USA) as follows: 3 min at 95 °C or denaturation, followed by 40 cycles of 7 s at 95 °C for denaturation, 10 s at 57 °C for annealing, and 30 s at 72 °C for extension. All gene expression analyses were performed using three independent biological replicates. The relative expression levels for each gene were normalized to the expression level of 18 S rRNA (internal reference gene), which was calculated from cycle threshold values using the 2^−ΔΔCt^ method^62^. The sequences of specific primers used for qRT-PCR are listed in Supplementary Table S9.

### Data Availability

The raw data generated in this study were deposited in the NCBI Sequence Read Archive (SRA, http://www.ncbi.nlm.nih.gov/Traces/sra) with the following accession numbers: SRR5658201-SRR5658204. The CGMMV virus source was preserved on bottle gourd plants by the Plant Virus Laboratory of the Plant Protection College, Shenyang Agricultural University, China. The sequence of this isolate (CGMMV-lnxg), which served as viral inoculums, was deposited in the GenBank with the accession number KY040049.

## Electronic supplementary material

Supplementary Figure 1

Supplementary tables S1–S9
