# Supplementary material for: Transcriptome analysis of watermelon (Citrullus lanatus) fruits in response to Cucumber green mottle mosaic virus (CGMMV) infection
Source: Sci Rep. 2017 Dec 1;7:16747. doi: 10.1038/s41598-017-17140-4 (PMC5711961; doi:10.1038/s41598-017-17140-4)
Supplement: Supplementary file 1 — Supplementary Figure 1 [file 41598_2017_17140_MOESM1_ESM.doc]

**Transcriptome analysis of watermelon (*****Citrullus lanatus*) fruits in response to *Cucumber green mottle mosaic virus* (CGMMV) infection**

# Xiaodong Li^1^, Mengnan An^1^, Zihao Xia^1^, Xiaojiao Bai^1^，and Yuanhua Wu^1,*^

^1^Plant Virus Laboratory of Plant Protection College, Shenyang Agricultural University, Shenyang 110866, China

*[wuyh7799@163.com](mailto:wuyh7799@163.com)

**Supplementary Info**

Supplementary Figure 1


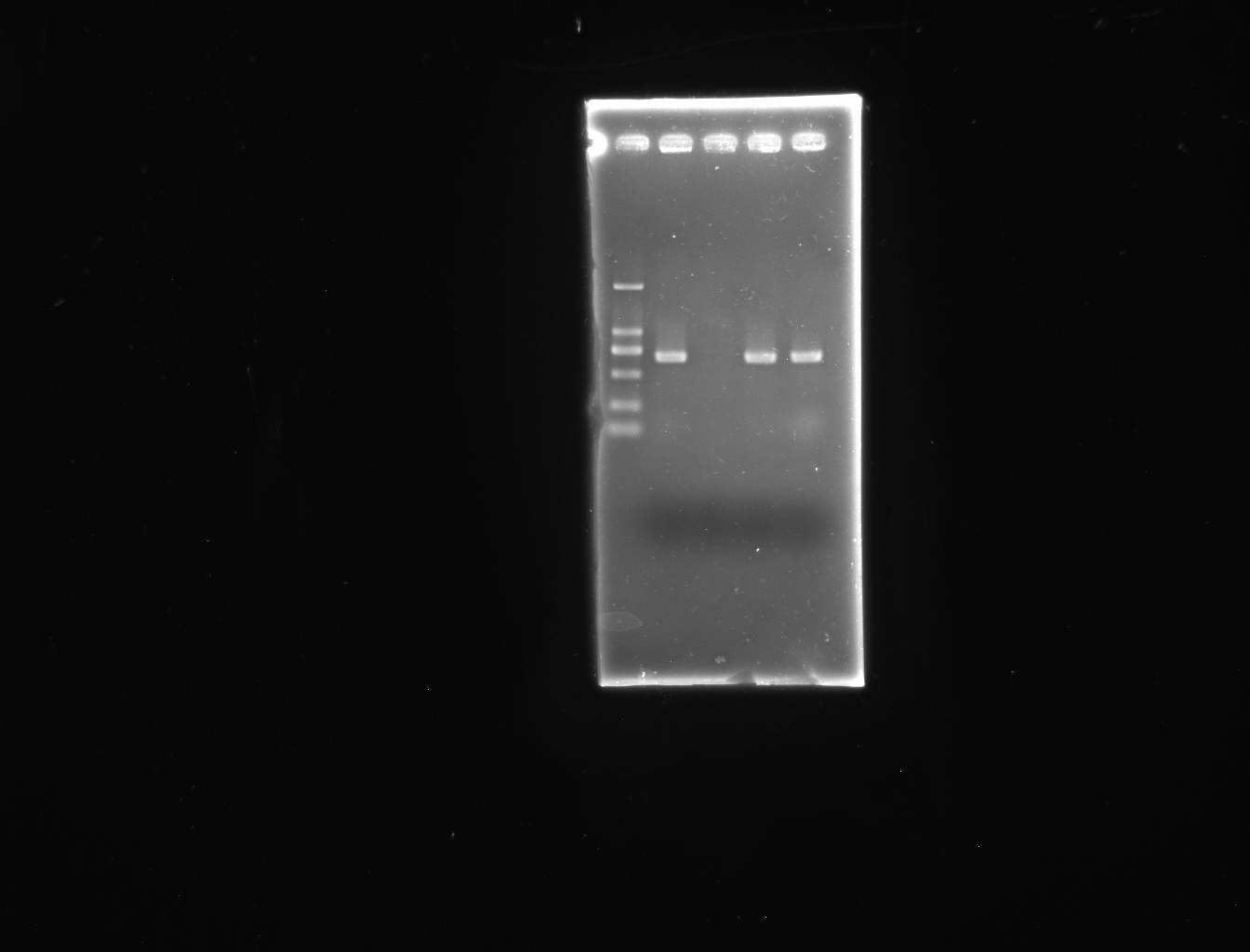


M 1 2 3 4

2, 000 bp

1, 000 bp

750 bp

500 bp

200 bp

100 bp

662 bp

**Supplementary Figure 1**. The full-length agarose gel electrophoresis of RT-PCR detection results of watermelon fruits with CGMMV specific primers (F: 5’-ATGGCTTACAATCCGATCAC-3’; R: 5’-TGGGCCCCTACCCGGGGA-3; the target fragment includes the complete CP gene of CGMMV). M indicates the DL 2, 000 DNA Marker. The DNA molecular weights of each band are shown on the left of the gel. Lane 1 and 2 indicate CGMMV-inoculated or mock-inoculated watermelon flesh, respectively. Lane 3 and 4 are both positive controls, which are from bottle gourd leaves (used as viral inoculums in this study). Only Lane 3 was retained in the **Figure 1c** of the main text. The expected PCR products are 662 bp and the corresponding bands are indicated by a red arrow on the right of the gel.
